# Serpina3n accelerates tissue repair in a diabetic mouse model of delayed wound healing

**DOI:** 10.1038/cddis.2014.423

**Published:** 2014-10-09

**Authors:** I Hsu, L G Parkinson, Y Shen, A Toro, T Brown, H Zhao, R C Bleackley, D J Granville

**Affiliations:** 1 Department of Pathology and Laboratory Medicine, University of British Columbia, Vancouver, British Columbia, Canada; 2 Centre for Heart Lung Innovation, St. Paul's Hospital, University of British Columbia, Vancouver, British Columbia, Canada; 3 Department of Biochemistry, University of Alberta, Edmonton, Alberta, Canada

## Abstract

Chronic, non-healing wounds are a major complication of diabetes and are characterized by chronic inflammation and excessive protease activity. Although once thought to function primarily as a pro-apoptotic serine protease, granzyme B (GzmB) can also accumulate in the extracellular matrix (ECM) during chronic inflammation and cleave ECM proteins that are essential for proper wound healing, including fibronectin. We hypothesized that GzmB contributes to the pathogenesis of impaired diabetic wound healing through excessive ECM degradation. In the present study, the murine serine protease inhibitor, serpina3n (SA3N), was administered to excisional wounds created on the dorsum of genetically induced type-II diabetic mice. Wound closure was monitored and skin wound samples were collected for analyses. Wound closure, including both re-epithelialization and contraction, were significantly increased in SA3N-treated wounds. Histological and immunohistochemical analyses of SA3N-treated wounds revealed a more mature, proliferative granulation tissue phenotype as indicated by increased cell proliferation, vascularization, fibroblast maturation and differentiation, and collagen deposition. Skin homogenates from SA3N-treated wounds also exhibited greater levels of full-length intact fibronectin compared with that of vehicle wounds. In addition, GzmB-induced detachment of mouse embryonic fibroblasts correlated with a rounded and clustered phenotype that was prevented by SA3N. In summary, topical administration of SA3N accelerated wound healing. Our findings suggest that GzmB contributes to the pathogenesis of diabetic wound healing through the proteolytic cleavage of fibronectin that is essential for normal wound closure, and that SA3N promotes granulation tissue maturation and collagen deposition.

Diabetic skin ulcers are non-healing, chronic wounds that pose a major health burden to society.^1^ Up to a quarter of diabetic patients will develop these ulcers in their lifetime and as a result, nearly a fifth of these diabetic patients will require non-traumatic lower limb amputations.^2^ Numerous advanced treatment options for the management of diabetic ulcers have been explored, including bioengineered skin substitutes, hyperbaric oxygen therapy and negative pressure dressings.^3, 4, 5^ However, they have largely been unsuccessful. As such, a combination of lifestyle modification, pressure off-loading, local surgical or larval debridement and infection control continue to be the standard recommended treatment strategy.^6^

Wound healing is a complex process that involves overlapping and sequential phases involving haemostasis, inflammation, granulation tissue formation and tissue remodelling. For wounds to heal timely and properly, there must be a fine balance of interaction between various cell types, cytokines, growth factors, proteases and extracellular matrix (ECM) components. However, in diabetic patients, the normal continuum of wound healing is disrupted, and wounds enter a chronic, non-healing state characterized by persistent inflammation, enhanced proteolytic activity and impaired ECM deposition.^7^ The roles of various proteases, primarily matrix metalloproteinases (MMPs), have been extensively studied. Although MMPs were once believed to be the major culprits in impaired wound healing, it is now recognized that they are essential for normal wound healing by modulating inflammation, angiogenesis and tissue remodelling.^8, 9, 10^ Therefore, other proteases may also contribute to the pathogenesis of chronic wound healing.

Granzyme B (GzmB) is a cytotoxic serine protease that is often viewed exclusively as a pro-apoptotic serine protease that is released from cytotoxic lymphocytes, along with the pore-forming, molecule perforin, to induce cell death.^11^ However, because GzmB can be induced in other types of immune and non-immune cells that often do not express perforin and/or do not form immunological synapses with target cells, there is emerging evidence supporting the paradigm that GzmB can accumulate and function in the extracellular milieu.^12^ In support, many ECM proteins are GzmB substrates and the consequences of such cleavage may be implicated in many diseases associated with aging and/or chronic inflammation such as abdominal aortic aneurysm (AAA), skin aging, atherosclerosis and wound healing using GzmB knockout mice.^13, 14, 15, 16^ Fibronectin is one such ECM glycoprotein that has an important role in cell attachment, differentiation and migration during wound healing and is cleaved by GzmB.^17^

The serine protease inhibitors, also known as serpins, are the largest protease inhibitor super family and are divided into 16 clades.^18^ Serpina3n (SA3N), which is part of the SERPINA clade, is the mouse orthologue of the human anti-chymotrypsin (ACT) and has been identified as an inhibitor of both human and mouse GzmB *in vitro*.^19, 20, 21^ The human ACT is encoded by only one gene; however, extensive diversification and duplication in mice have resulted in 13 related serpina genes clustered at chromosome 12F1.^19^ Out of these, SA3N was identified to be the only extracellular inhibitor of GzmB.^21^ A previous study by our group had demonstrated efficacy for SA3N as an *in vivo* GzmB inhibitor in a murine model of AAA.^20^

The purpose of this study was to determine whether local inhibition of extracellular GzmB could accelerate wound closure in a genetically induced type-II diabetic mouse model of delayed wound healing. As many of the proteins in the ECM involved in wound healing are subject to cleavage by GzmB, we propose that SA3N accelerates wound healing by inhibiting protease-related ECM degradation.

## Results

### GzmB is elevated and differentially expressed at the wound edges and granulation tissues of diabetic wounds

**Figure 1:**
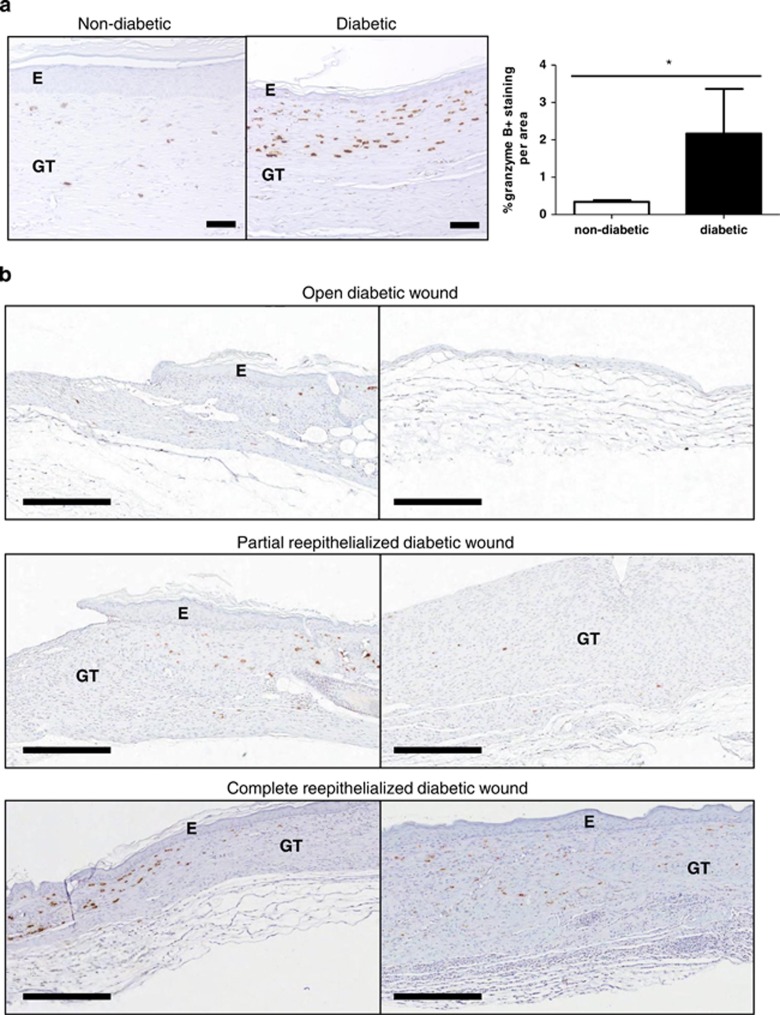
Expression of GzmB at edges and middle of diabetic wounds. (**a**) Using immunohistochemistry, GzmB expression, denoted by brown staining, was higher in completely re-epithelialized diabetic wounds in comparison with non-diabetic wounds. (**b**) GzmB expression was found to be localized to wound edges (left panels) and granulation tissues (right panels) in open (at day 12), partially re-epithelialized (at day 18) and completely re-epithelialized (at day 35) diabetic wounds. (E denotes epidermis and GT denotes granulation tissue. Error bars represent mean±S.D. *P*-value was calculated using unpaired Student *t*-test. *P<0.05. Black scale bars=50 *μ*m for (**a**) and 300 *μ*m for (**b**).

Chronic inflammation is a hallmark of diabetic wounds. As GzmB may accumulate in the extracellular milieu during chronic inflammation, GzmB levels were assessed in wounded tissues. GzmB levels in diabetic wounds were significantly higher than the levels found in non-diabetic wounds (*P*=0.0225; Figure 1a). The expression of GzmB in diabetic wounds at different healing stages was assessed by immunohistochemistry (Figure 1b). In an open wound, GzmB expression was low and minimal GzmB was localized to wound edges. In a partially re-epithelialized wound, GzmB expression was moderate and localized to mainly wound edges, with diffuse staining throughout the granulation tissues. In a fully re-epithelialized wound, GzmB expression was abundant and scattered throughout the wound edges and granulation tissues. These results confirmed that GzmB is present in the different phases of wound healing.

### SA3N accelerates diabetic wound closure

**Figure 2:**
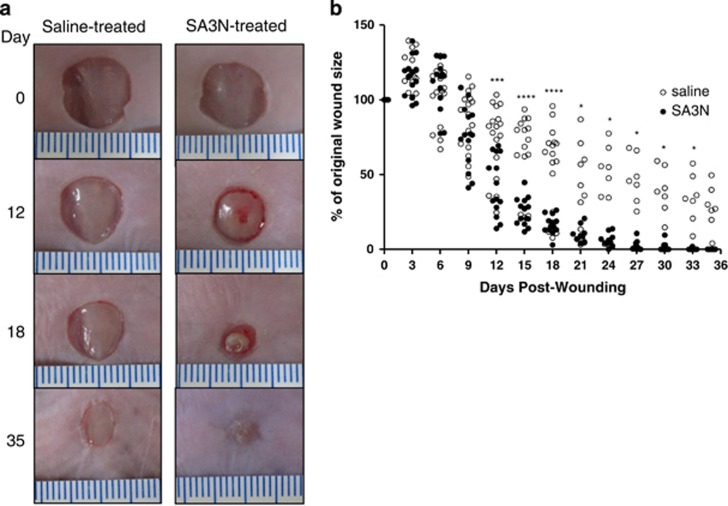
SA3N accelerated diabetic wound closure. (**a**) Representative digital images of non-diabetic and diabetic mice wounds captured over 35 days wound-healing period. (**b**) Comparison of the percentages of the open wound size over 35 days healing period between vehicle- (*n*=17) and SA3N-treated diabetic mice (*n*=14), showing that SA3N-treated wounds healed significantly faster than vehicle-treated wounds and that the healing rate was similar to that of non-diabetic wounds. (Open circles represent replicates of saline-treated wounds and closed circles represent replicates of SA3N-treated wounds. *P*-values were calculated using unpaired Student *t*-test. *P<0.05, ***P<0.001, ****P<0.0001 between vehicle and SA3N-treated wounds.)

To investigate whether SA3N accelerates wound closure, digital images of wounds were captured during dressing changes at a fixed distance and angle for planimetry measurements. SA3N significantly accelerated wound closure compared with vehicle control over 35 days (Figure 2). In all animals, wound sizes initially expanded. The difference in wound closure between the two treatment groups was significantly different by day 12, as the SA3N-treated wounds began to reveal provisional matrix formation on top and surrounding the original wounds as they closed. By day 18, the wound size relative to day 0 was significantly reduced in SA3N-treated animals (16.4±6.1% *n*=14) *versus* vehicle-treated animals (53.7±28.7% *n*=17).

### SA3N increases re-epithelialization, contraction and granulation tissue formation in diabetic wounds

**Figure 3:**
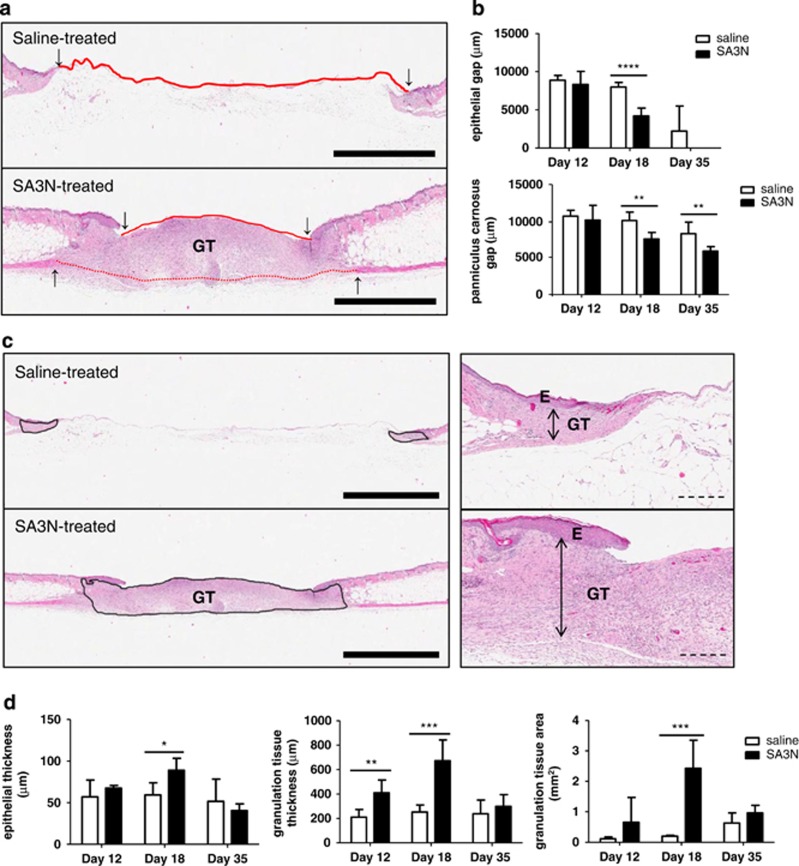
SA3N increased wound re-epithelialization, contraction and granulation tissue formation in diabetic wounds. (**a**) Representative histological images of skin sections from day 18 wounds treated with vehicle and SA3N. Downward arrows and red solid lines denoted ends of epithelial tongue. Upward arrows and red dotted lines denoted ends of PC layer. (**b**) The distances between the ends of the epidermis and/or the PC were significantly less in SA3N-treated wounds at day 18. (**c**) Representative histological images of skin sections from day 18 wounds treated with vehicle and SA3N. Area outlined by black line represented granulation tissue, downward arrows represented epithelial thickness and double arrows represented granulation tissue thickness. (**d**) Increases in both epithelial and/or granulation tissue thicknesses, and overall granulation tissue areas, were observed in SA3N-treated wounds at days 12 and 18. (E denotes epidermis, D denotes dermis, H denotes hypodermis, PC denotes PC and GT denotes granulation tissue. Error bars represent mean±S.D. *P*-values were calculated using unpaired Student *t*-test. *P<0.05, **P<0.01, ***P<0.001, ****P<0.0001. Black scale bars=2 mm. Dotted scale bars=300 *μ*m.)

During the granulation tissue formation phase of wound healing, both re-epithelialization and contraction facilitate wound closure. Bisected wound tissues collected from all animals at days 12, 18 and 35 were sectioned and stained with haematoxylin and eosin (H&E) for histological analyses. The distances between the leading edges of the epithelial tongues and the panniculus carnosus (PC) muscle layers were both significantly shorter in the SA3N-treated animals by day 18 (*P*⩽0.0001 and *P*=0.003; Figures 3a and b). At day 35, there was no difference in the distances of epithelial tongues between the two treatment groups, but the distance between the PC muscles layers were still significantly shorter in the SA3N-treated animals. Furthermore, by day 18, the epithelial and granulation tissue thicknesses and granulation tissue areas of SA3N-treated wounds were significantly greater than that in vehicle-treated wounds (*P*=0.012, *P*=0.0008 and *P*=0.0007; Figures 3c and d). However, by day 35, the differences in the epithelial and granulation tissue thicknesses and granulation tissue areas between the vehicle- and SA3N-treated wounds were not significant.

### SA3N promotes maturation of granulation tissues in diabetic wounds

**Figure 4:**
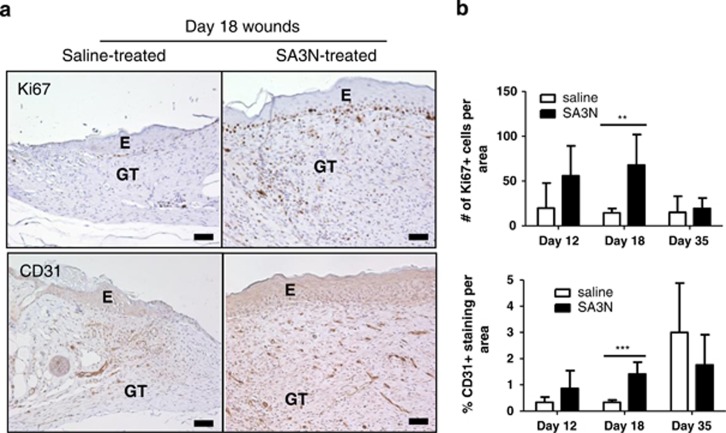
SA3N promoted granulation tissue maturation of diabetic wounds. (**a**) Immunohistochemical staining of skin sections for Ki67 (a marker of proliferation cells) and CD31 (a marker of vasculature), where positive staining is brown. (**b**) Greater number and positive staining of proliferating cells and blood vessels were observed in wounds treated with SA3N at day 18. (E denotes epidermis and GT denotes granulation tissue. Error bars represent mean±S.D. *P*-values were calculated using unpaired Student *t*-test. **P<0.01, ***P<0.001. Black scale bars=50 *μ*m.)

The formation and maturation of granulation tissue is a highly active process. Many different cell types contribute to matrix synthesis, organization and wound repair. Skin sections from the middle of wounds were stained for Ki67 and CD31− markers for cellular proliferation and vasculature, respectively (Figure 4a). At day 18, the granulation tissues of SA3N-treated wounds exhibited significantly greater expression of Ki67 and CD31 as shown by Figure 4b (*P*=0.0086 and *P*=0.0008). Furthermore, there was no difference in the immunohistochemical staining of Ki67 and CD31 between the wounded tissues at day 35.

### SA3N stimulates fibroblast deposition and attachment in mouse embryonic fibroblasts

**Figure 5:**
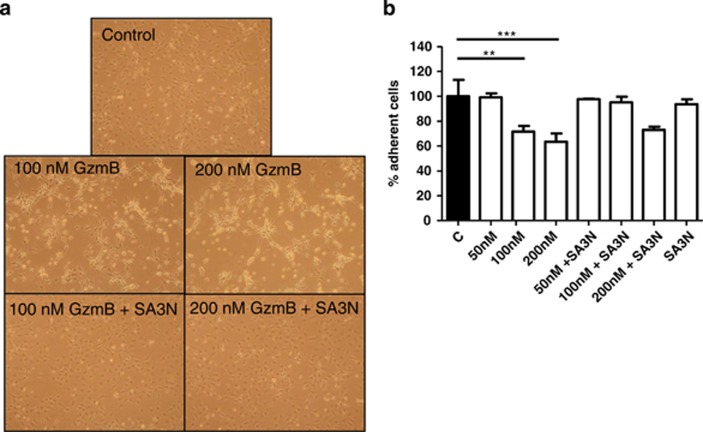
GzmB promoted detachment of mouse embryonic fibroblasts. (**a**) Representative images of mouse embryonic fibroblasts when treated with GzmB±SA3N. In vehicle and GzmB+SA3N wells, fibroblasts were well attached and spread out with many filopodia protrusions. In GzmB-only wells, fibroblasts appeared detached. (**b**) GzmB reduced adherence of mouse embryonic fibroblasts in a dose-dependent manner and co-treatment with SA3N reversed the effect. (Error bars represent mean±S.D. *P*-values were calculated using one-way ANOVA with Dunnett's multiple comparison test. **P<0.01, ***P<0.001.)

As GzmB is capable of cleaving ECM substrates that are important in cell attachment, ECM integrity and deposition, the effect of GzmB on fibroblast viability was examined. On exogenous GzmB treatment, fibroblasts exhibited a marked change in morphology, with many cells rounding up and detaching over the incubation period compared with vehicle cells, which appeared well attached and spread with many filopodia protrusions (Figure 5a). The treatment effect of GzmB was abrogated via co-treatment with SA3N, with the morphology of cells comparable to those in the vehicle group (Figure 5a). In addition to the qualitative assessment, we also quantified the relative percentage of adherent cells remaining after GzmB treatment by using a MTS viability assay. Addition of GzmB resulted in a dose-dependent decrease in adherent fibroblasts, whereas co-incubation with SA3N protected against cell detachment-mediated cell death (Figure 5b).

### SA3N promotes fibroblast maturation and collagen deposition in diabetic wounds

**Figure 6:**
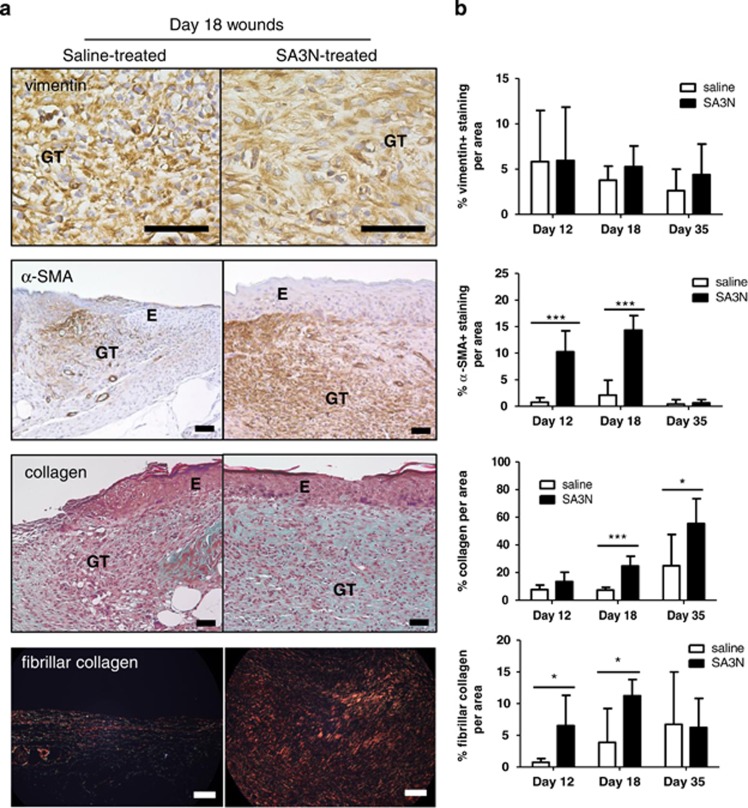
SA3N promoted fibroblast maturation and collagen deposition in the granulation tissues of diabetic wounds. (**a**) The first two rows of panels are representative images of immunohistochemical staining of skin sections for vimentin (a marker of fibroblasts of mesenchymal origin) and α-SMA (a marker of myofibroblasts), where positive staining was coloured brown. The fibroblasts present in vehicle-treated wounds appeared more circular, whereas the fibroblasts in SA3N-treated wounds appeared elongated and spindle-like. The third row of panels represents Masson's trichrome staining of skin sections where keratin and muscle fibres appeared red and collagen appeared blue–green. The last row of panels represents picrosirius red staining of skin sections under polarized light, and the fibrillar collagen appeared red. (**b**) There was no difference in levels of vimentin between the two treatment groups at day 18. Greater positive staining and percentage of myofibroblasts, collagen and fibrillar collagen were observed in wounds treated with SA3N at day 18. (E denotes epidermis and GT denotes granulation tissue. Error bars represent mean±S.D. *P*-values were calculated using unpaired Student *t*-test. *P<0.05, ***P<0.001. Black scale bars=50 *μ*m and white scale bars=100 *μ*m)

Following upon the observations *in vitro*, fibroblast morphology and maturation was investigated in vehicle and SA3N-treated wounds. Skin sections from diabetic wounds were assessed for vimentin and alpha-smooth muscle actin (α-SMA), a marker for fibroblast of mesenchymal origin and myofibroblasts (Figure 6). A morphological difference was observed in the appearance of the fibroblasts, where the fibroblasts present in the vehicle wounds were rounded up and clustered. Alternately, fibroblasts present in the SA3N-treated wounds exhibited a similar resemblance to that observed *in vitro*, noted by the flat, spread out, elongated and spindle-like shape (Figure 6a). Immunohistochemical analyses revealed there was no notable difference in the relative expression of vimentin at day 18. However, granulation tissues of SA3N-treated wounds at day 18, but not day 35, exhibited significantly greater expression of α-SMA (*P*=0.0001).

Collagen content was assessed using Masson's trichrome staining (Figure 6). SA3N-treated wounds had significantly greater green–blue positive staining for collagen in comparison with vehicle wounds on days 18 (*P*=0.0007). We also specifically examined the levels of types-I and -III fibrillar collagen by using picrosirius red staining and found that the percentage of fibrillar collagen per area was significantly greater in SA3N-treated wounds at day 18 (*P*=0.0239). This was an indication of more mature granulation tissues by day 18 in the SA3N-treated wounds when the provisional matrices had largely formed.

### SA3N inhibits degradation of fibronectin *in vitro* and degradation of fibronectin in diabetic wounds

**Figure 7:**
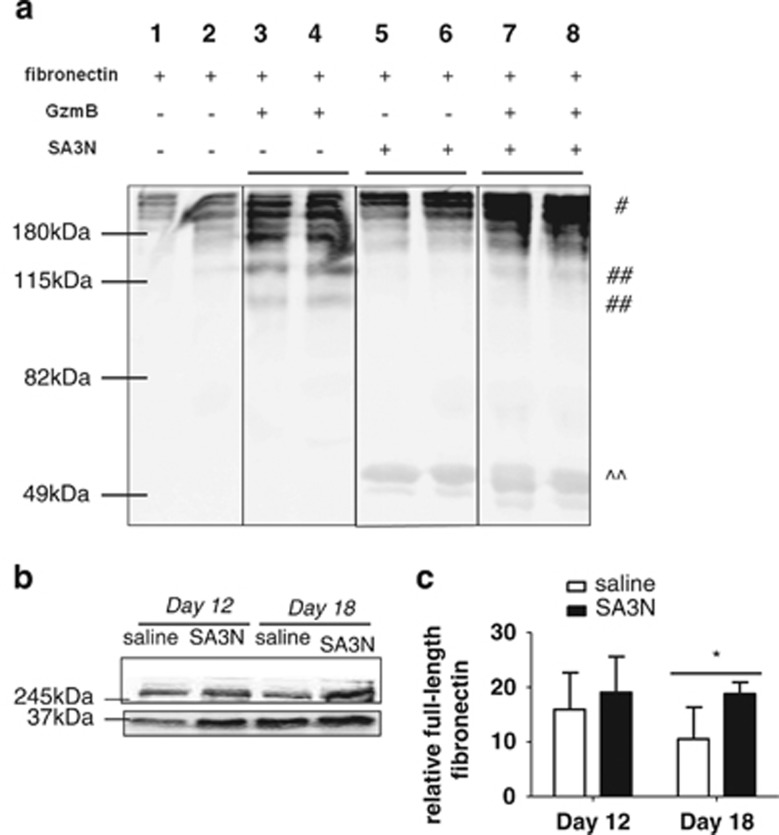
SA3N inhibited degradation of GzmB *in vitro* and degradation of fibronectin in diabetic wounds. (**a**) Wells were coated with fibronectin and incubated with GzmB and/or SA3N. Supernatants were subsequently collected and cleavage fragments were assessed by western blot. In all lanes, the full-length fibronectin spontaneously released over incubation period was observed and denoted by ^#^. In lanes 3 and 4, GzmB-mediated fibronectin fragments were observed and the resulting cleavage fragments were denoted by ^##^. Inhibition of mouse GzmB with SA3N resulted in fewer fragments as seen in lanes 7 and 8. The presence of SA3N was denoted by ^^ in lanes 5, 6, 7 and 8. (**b**) Representative western blot probing for fibronectin in skin homogenates collected from wounds treated with vehicle and SA3N. The top band is full-length fibronectin and the bottom is loading control, GAPDH. (**c**) The full-length fibronectin content was significantly greater in homogenates of SA3N-treated wounds, indicating less cleavage when normalized to GADPH and analysed by densitometry. (Error bars represent mean±S.D. *P*-values were calculated using unpaired Student *t*-test. *P<0.05.)

In the present study, mouse GzmB cleaved mouse fibronectin and the cleavage of mouse fibronectin by mouse GzmB was inhibited by SA3N as depicted by fewer fragments observed in Figure 7a. This also confirmed that SA3N can inhibit mouse GzmB-mediated cleavage of mouse fibronectin. To examine fibronectin content and possibly fibronectin fragmentation *in vivo*, we homogenized wound tissues from vehicle- and SA3N-treated wounds, and subsequently, probed for fibronectin by western immunoblotting. There was no difference in full-length fibronectin content between the two treatment groups on day 12 (Figure 7b). However, SA3N-treated wounds exhibited significantly more full-length fibronectin after normalizing to glyceraldehyde 3-phosphate dehydrogenase (GAPDH) when compared with vehicle-treated wounds at day 18, thereby indicating that SA3N protected the degradation of fibronectin (*P*=0.0374; Figure 7c).

## Discussion

Since the discovery of GzmB over a quarter of a century ago, most studies and understanding of GzmB biology has focussed on its pro-apoptotic role.^22,23^ However, emerging evidence in recent years suggests a clear role for extracellular GzmB in a number of immunopathologies to the point whereby it is now generally accepted that GzmB may function in the extracellular milieu and that its accumulation in the extracellular environment is implicated in many chronic inflammatory diseases.^24, 25, 26, 27^ Currently, no known endogenous extracellular GzmB inhibitor exists in humans, so the elevated levels of GzmB observed in bodily fluids may contribute to dysregulated proteolysis.^28, 29, 30, 31, 32, 33, 34^ As non-healing wounds feature chronic inflammation and elevated proteolytic activity, we speculated that GzmB may contribute to the pathogenesis of chronic wound healing, as many substrates of GzmB are ECM proteins essential for proper wound healing.^20,35,36^

Due to aging demographics and an increasing diabetic patient population, the incidence of chronic non-healing wounds is on the rise.^37^ In relevance to the possible role of GzmB in age-related chronic wounds, a previous study by our group demonstrated that apolipoprotein-knockout mice fed a high-fat diet for 37 weeks exhibited delayed wound closure that was not present in apolipoprotein-GzmB double knockout mice due, in part, to GzmB-mediated degradation of two ECM proteins, fibronectin and vitronectin.^16^ To our knowledge, it was the first study implicating a pathogenic role for GzmB in chronic wound healing and suggested that GzmB inhibition may be a viable therapeutic target for chronic wounds. The main purpose of this study was to determine whether GzmB contributes to the pathogenesis of chronic wound healing in individuals with diabetes. Specifically, we utilized a genetically induced type-II diabetic mouse model of delayed wound healing to mimic the underlying hyperglycemic condition associated with diabetic patients. Instead of a systemic knockout study, we investigated whether local inhibition of extracellular GzmB could accelerate wound healing in this model by using the serine protease inhibitor, SA3N, that inhibits GzmB.

The interaction between cells and the ECM is essential in creating a microenvironment that promotes normal acute wound healing.^38^ Abnormal levels of proteases and fragmentation of ECM proteins can disrupt ECM integrity and lead to the development of chronic wounds. The present study demonstrated that in comparison with non-diabetic wounds, diabetic wounds exhibited higher GzmB expression in mice. There have been various studies showing elevated levels and increased proteolytic activity of MMPs in human chronic wound fluid, including MMP-2, MMP-8 and MMP-9.^39, 40, 41^ In addition to MMPs, groups also reported elevated activity of other serine proteases in human chronic wound fluid, including plasmin, neutrophil elastase and cathepsin G.^42, 43, 44^ Another hallmark of chronic wound fluid is fibronectin fragmentation, and fragment sizes ranging from 20 to 202 kDa have been reported by previous studies.^42,44, 45, 46, 47, 48, 49^ The study by Rao *et al.*^47^ attributed serine proteases to be the main contributor of fibronectin fragmentation in chronic wound fluid samples. In our study, we confirmed that fibronectin was cleaved by GzmB and that this cleavage was inhibited when GzmB was incubated together with SA3N. In support of our *in vitro* findings, homogenized tissues from the SA3N-treated wounds exhibited a greater level of full-length fibronectin when compared with vehicle-treated wounds. Although fibronectin is cleaved by GzmB, the site of cleavage is still currently unknown.^16,35,50^ It has been suggested that cleavage occurs at the RGD (Arg-Gly-Asp) integrin-binding site as GzmB cleavage of vitronectin occurs at the RGD site.^35^

The present study demonstrated that wound closure was significantly accelerated in diabetic mice treated with SA3N. This was observed beginning at day 12 when the wounds appeared less inflamed as initial haemostasis and inflammation phases had subdued, and the granulation tissue formation phase had begun with formation of the typical white-coloured provisional matrix as observed in the SA3N-treated wounds. During granulation tissue formation, fibroblasts secrete proteoglycans and collagen. Fibronectin is a scaffolding protein and the attachment of fibroblasts to the glycoprotein is mediated through integrin, including α4β1 and α5β1.^51^ Therefore, fibronectin fragmentation can impair fibroblast attachment and maturation. In our study, a reduction in fibroblast spreading and attachment was observed in GzmB-challenged murine fibroblasts, which was similar to the morphology of fibroblasts observed in vehicle-treated mouse wounds. Conversely, cultured mouse fibroblasts that were treated with both GzmB and SA3N, as well as fibroblasts of SA3N-treated wounds, were well attached with extensive spreading. The observations associated with impaired fibroblasts and that fibronectin fragmentation could be contributing to functional defects in fibroblasts are supported by previous studies.^35,46,48^ Stanley *et al.*^46^ noted that attachment efficiency and migration of primary human gingival fibroblasts and fibrosarcoma cells decreased when coated on a matrix containing fragmented fibronectin. They also observed that cells seeded on fragmented fibronectin exhibited less filopodia spreading and formed more clusters than cells plated on full-length intact fibronectin.^46^ Grinnell *et al.*^48^ also reported similar findings where they observed baby hamster kidney fibroblasts rounding up when the cells were treated with chronic wound fluid containing fragmented fibronectin and vitronectin. In addition, Buzza *et al.*^35^ have also shown that GzmB is responsible for cell detachment and the reduced spreading and migration of many different cell lines, including primary human vascular endothelial and human breast adenocarcinoma cells.

The formation, maturation and deposition of the ECM in the granulation tissues ensure that there is a proper tissue restoration in the subsequent remodelling phase, as differentiation of fibroblasts to myofibroblasts is critical for wound contraction and production of collagen fibres. The granulation tissues of SA3N-treated wounds featured elevated levels of myofibroblasts and increased collagen deposition when compared with vehicle-treated wounds at day 18. The differentiation of fibroblasts to myofibroblasts is largely dependent on three factors: TGFβ-1, the ED-A domain of fibronectin and mechanical tension.^52,53^ Typically, cellular fibronectin is synthesized without an ED-A domain.^54^ However, during wound healing, endothelial cells and fibroblasts synthesize a splice variant of the cellular fibronectin containing an extra ED-A domain.^55^ For differentiation of fibroblasts to occur, TGFβ-1 interacts with the ED-A domain of fibronectin to trigger downstream signalling and induction to a myofibroblast-like phenotype by increasing expression of *α*-SMA.^55,56^ The increase in α-SMA expression supported the observation that wound closure was accelerated in SA3N-treated mice, as wound contraction is one of the two means by which wounds close, with myofibroblasts expressing α-SMA being largely responsible. In addition, it is possible that GzmB cleavage of fibronectin may disrupt the interaction between TGF*β*-1 and the ED-A domain, thus leading to the decreased myofibroblasts and collagen deposition that was observed in the granulation tissues of vehicle wounds, as these wounds also feature increased fibronectin degradation.

Greater granulation tissue formation was observed in SA3N-treated wounds and these tissues were characterized by increased cellular proliferation and blood vessel formation by day 18. The increase in Ki67 expression highlighted the proliferative state of the granulation tissues. Decreased proliferative capacity has been observed in fibroblasts isolated from human chronic wound samples, indicating that many of the fibroblasts are in a senescent state.^57, 58, 59, 60^ The increase in CD31 expression, indicative of *de novo* blood vessel formation, suggests that treatment also improves tissue perfusion compared with controls. Previously, we demonstrated that GzmB-mediated fibronectin cleavage contributes to impaired angiogenesis and vascular permeability through a process involving reduced endothelial adherence, tubule formation and the release of fibronectin-sequestered VEGF.^50,61^ As such, inhibition of GzmB activity may promote vascularization in chronic wounds and facilitate healing. By day 35, the number of proliferating cells, blood vessels, mesenchymal cells and myofibroblasts had decreased from day 18 in the SA3N-treated wounds and that there was no difference between the saline- and SA3N-treated wounds. This was expected by the time the wound closed, as the wounds transitioned into the remodelling phase of wound healing. The resolution of the hyperactive granulation phase demonstrates a robust wound-healing process with SA3N treatment.

A limitation to the current study is that, although SA3N is a potent inhibitor of GzmB and only known inhibitor of extracellular GzmB that has been used *in vivo*, SA3N can also inhibit chymotrypsin, neutrophil elastase and cathepsin G.^19, 20, 21^ SA3N has been utilized previously in an animal model of AAA where the rupture rates and ECM remodelling was similar to that observed in using GzmB knockout mice.^13,20^ Although the present results, in combination with previous studies using GzmB-KO mice in impaired wound-healing models, suggest that GzmB impairs wound healing, further studies using a potent GzmB-specific inhibitor are required to fully extract the role of GzmB in impaired healing.

In summary, using a genetically induced type-II diabetic model of delayed wound healing, we demonstrated that SA3N accelerates wound healing by promoting granulation tissue maturation, inhibition of fibronectin cleavage and stimulation of collagen deposition in remodelling tissues. The results from this study suggest that GzmB might be contributing to the pathogenesis of diabetic wound healing, and that GzmB is a relevant therapeutic target in wound management therapy.

## Materials and Methods

### Animals

The animal studies were conducted in accordance with the procedure guidelines approved by the University of British Columbia Animal Care Committee. All mice used in the studies were purchased from the Jackson Laboratories (stock number 000642; Bar Harbor, ME, USA) at 7 weeks. All mice used were from a C57BLKS/J background strain, male and either homozygous or heterozygous for the spontaneous mutation in the leptin receptor. The mice homozygous for the leptin receptor mutation were considered to be diabetic. The mice heterozygous for the leptin receptor mutation were considered to be the non-diabetic littermates. All mice were fed standard laboratory chow (equal parts of PicoLab Mouse Diet 20 #5058 and PicoLab Rodent Diet 20 #5053 from LabDiet, Richmond, IN, USA) and water *ad libitum*.

Two days before the wound-healing surgery, all mice were fasted for 4 h and fasting blood glucose levels of the mice were determined with OneTouch Ultra Blood Glucose Meter (LifeScan, New Brunswick, NJ, USA). Only mice with fasting blood glucose levels >200 mg/dl were considered diabetic. The hair on the backs of the animals were removed by shaving with hair clippers followed by treatment with Nair (Church and Dwight, Ewing, NJ, USA) for 90 s. Finally, only mice with no visible active hair follicles were subjected to surgery.

### Wound-healing surgery

All mice were anaesthetized using a mixture of isoflurane and oxygen on a warm heating pad to prevent hypothermia. Once the mice had reached proper anaesthetic depth, eye lube was applied and buprenorphine (0.05 mg/kg; McGill University, Montreal, QC, Canada) was administered subcutaneously. The skin area to be wounded was then prepared by swabbing with betadine and two washes of 70% ethanol. After prepping, a 10-mm diameter punch biopsy (Acuderm, Fort Lauderdale, FL, USA) was used to outline a wound pattern and a full-thickness skin excision (including the PC muscle layer) was created on the mid-lower back of the mice using sterilized surgical scissors. Once wounded, digital pictures of the wound area were captured at a fixed distance and angle, and wound area was covered by a transparent Tegaderm dressing (3M, Minneapolis, MN, USA). All surgical animals were allowed to recover and were housed individually to prevent the removal of dressings by other animals.

### Treatment

Wound areas were cleaned by washing with sterile vehicle and replaced with new Tegaderm every 3 days. At this time, digital pictures of the wound area were captured, with ruler placed below, for planimetry measurements and also taken every 3 days until day of killing (either days 12, 18 or 35 post wounding). In addition, all mice were treated immediately after wounding and every 3 days until day of kiiling. The mice were divided into two treatment groups and received either sterile vehicle or SA3N (at a concentration of 35 ng/*μ*l diluted with sterile vehicle; generous gift from Dr. Chris R Bleackley of University of Alberta, Edmonton, AB, USA) in a 100 *μ*l volume. On days 0, 3, 6 and 9, the treatment was administered topically by injecting directly underneath the Tegaderm with an insulin syringe. Starting from day 12 and every treatment day onwards, vehicle or SA3N was administered subcutaneously by injecting into surrounding wound areas, either top and bottom or right and left.

### Tissue collection and processing

On the day of killing, mice were killed by carbon dioxide asphyxiation. A 7 mm × 7 mm representative piece of wounded tissue was collected and cut in half, bisecting the wound centre. The left half was immediately flask frozen in liquid nitrogen and stored at −80 °C. The right half was fixed in 10% phosphate-buffered formalin overnight, and transferred to 70% ethanol, before paraffin embedding for histological and immunohistochemical analyses.

### Histology and immunohistochemistry

Five-micron sections were deparaffinized and rehydrated in the following order for histological and immunohistochemical stainings: three washes of xylene, 100% ethanol, 90% ethanol, 70% ethanol and two washes of Tris-buffered vehicle (TBS). Slides were stained with H&E for evaluation of morphology, Masson's trichrome to detect collagen and picrosirius red to detect fibrillar collagen.

For immunohistochemistry, slides were heated in 80 °C citrate buffer (pH 6.0) for 10 min, cooled at room temperature for 30 min and quenched in 3% hydrogen peroxide for 10 min. These slides were then blocked with 10% goat serum mixture for 1 h at room temperature to reduce non-specific background staining. Subsequently, the slides were incubated with primary antibody prepared in a 10% goat serum mixture overnight at 4 °C. The primary antibodies used were a rabbit anti-mouse GzmB antibody (1:100; Abcam, Cambridge, MA, USA), a rabbit anti-mouse α-SMA antibody (1:2000; Abcam), rabbit anti-mouse CD31 antibody (1:200; Abcam) and rabbit ant-mouse Ki67 antibody (1:500; Cell Signaling Technology, Danvers, MA, USA). The following day, slides were incubated with biotinylated goat-anti rabbit secondary antibody (1:350; Vector Laboratories, Burlingame, CA, USA) prepared in a mixture of 5% goat serum for 30 min at room temperature, washed twice in TBS and incubated with Vectastain Elite ABC reagent (Vector Laboratories) for 30 min. Subsequently, the slides were washed twice in TBS-Tween 20 (TBST) and incubated with Vector DAB substrate (Vector Laboratories) for 5 min. Finally, the slides were then counterstained with haematoxylin for 30 s for visualization and quantification.

### Wound picture analyses

Digital pictures of the wound area were captured, with ruler placed below, on the day of wound-healing surgery and every 3 days until day of sacrifice. For all wounds, the open wound was normalized to wound size at day 0 and expressed as a percentage.

### Histological analyses

All quantification associated with H&E-stained slides were assessed by Aperio ImageScope version 11.1.2.760 (Aperio, Vista, CA, USA) and normalized to the percentage of original wound area. For analysis of epithelial and PC gaps, the two ends of each layer were identified and the distance between the two ends was measured. For measurement of epithelial thickness, the thickest distance between top and bottom of the epithelium was measured. For measurement of granulation tissue thickness, the thickest distance from top to bottom of the granulation tissue was used. For measurement of granulation tissue area, the whole granulation tissue area was traced. For quantification of collagen in Masson's trichrome-stained slides, all images were taken at × 20 magnification from the middle and/or the two edges of the wound. Using Image Pro Plus version 4.5.0.29 (Media Cybernetics, Rockville, MD, USA), the intensity RGB threshold was set for the blue–green positive colour of collagen and the number of positive pixels was counted and expressed as a percentage collagen per traced representative area. For quantification of fibrillar collagen in picosirius red-stained slides, all images were taken at × 20 magnification from the middle of the wound under polarized light. Using Image Pro Plus, the intensity RGB threshold was set for the reddish positive colour of fibrillar collagen and the number of positive pixels was counted and expressed as a percentage of fibrillar collagen per traced representative area.

### Immunohistochemical analyses

For quantification of all immunohistochemical staining, all images were taken either at × 20 or × 40 magnification from the middle and/or both edges of the wound. For Ki67 counting, representative areas from each image were defined and numbers of positive brown-staining cells per area were counted. For all the rest of the immunohistochemical analyses of GzmB, α-SMA and CD31, an intensity threshold was set for the brown colour and the number of positive pixels was counted and expressed as a percentage positive staining per traced representative area using Image Pro Plus.

### Fibronectin cleavage assay

The 48-well plates were coated with 3 *μ*g of mouse fibronectin (#92784, Abcam) per well diluted in PBS at 37 °C for 2 h and subsequently blocked with 1% bovine serum albumin for 30 min at 37 °C. The wells were treated with 50 nM mouse GzmB (Sigma-Aldrich, St. Louis, MO, USA)±100 nM SA3N at 37 °C overnight. The next day, supernatants were collected from each well and cleavage fragments were analysed by western blot.

### Mouse embryonic fibroblasts' detachment assay

3T3 mouse embryonic fibroblasts were plated to wells of a 96-well tissue culture plate (1.25 × 10^4^/well) in Dulbecco's Modified Eagle's Medium containing 10% fetal bovine serum and 1% penicillin–streptomycin, and allowed to adhere overnight in a 37 °C incubator with 5% carbon dioxide. Cells were washed with phosphate-buffered vehicle (PBS) and incubated in 100 *μ*l of serum-free media (SFM) containing 50, 100 or 200 nM GzmB±100 nM SA3N. Cells were incubated at 37 °C for 7 h, after which images of the cells in the plate were captured under phase contrast using a microscope camera. The media were aspirated and cells were washed once with PBS to remove non-adherent cells. Fresh SFM was replaced, and for quantification of remaining adherent cells, CellTiter AQueous One Solution MTS Cell Proliferation Assays (Promega, Madison, WI, USA) were performed by the addition of 20 *μ*l of MTS reagent and incubation for 3 h. Absorbance was read at 490 nm with a Magellan Tecan SaFire 2 plate reader (Tecan Group, Männedorf, Switzerland).

### Skin homogenization

Frozen skin sections were cut to 5 mm × 5 mm pieces and placed in a centrifuge tube containing a cold 5 mm stainless steel bead and 300 *μ*l of urea lysis buffer (50 mM Tris, pH 7.4, 7 M urea, 150 mM sodium chloride, 1% Triton) with 4 *μ*l of protease inhibitor cocktail (Sigma-Aldrich). The skin sections were homogenized in TissueLyser LT (Qiagen, Venlo, the Netherlands) at 50 Hz for 4 min, three times in total. In between each cycle, tubes were placed on ice for 30 sec to prevent overheating of the samples. After homogenization, samples were incubated on ice for 15 min and centrifuged at 4 °C for 10 min at 15 000 r.p.m. to separate proteins from pelleted cell debris. The supernatant was then transferred to a new tube containing 250 *μ*l PBS with 0.1% sodium dodecyl sulphate (SDS) and centrifuged at 4 °C again for 10 min at 15 000 r.p.m. to separate proteins from pelleted cell debris. The supernatant was transferred to Amicon Ultra Filter (Millipore, Billerica, MA, USA) and spun at 14 000 r.p.m. at 4 °C for 12 min, three times in total. The filtrate was discarded and 400 *μ*l PBS with 0.1% SDS was added after each spin to exchange the buffer. Total protein concentration of homogenized samples was measured and calculated using standard Bradford protein assays (Bio-rad, Hercules, CA, USA) such that samples were adjusted to ensure equivalent amounts of total protein for western blot.

### Western blots

For preparation of samples for western blot, either 25 *μ*l of supernatant (for fibronectin cleavage assay) or 16.5 *μ*g of homogenized protein (for fibronectin detection in wounded tissues) was added to 6 × Laemmli buffer (300 nM Tris-hydrogen chloride, pH 6.8, 600 mM dithiothreitol, 12% w/v SDS, 40% w/v glycerol, 0.25% w/v bromophenol blue) and denatured at 100 °C for 5 min. Boiled samples were run on a 10% SDS-polyacrylamide gel at 85 V for 15 min and 125 V for 90 min, and were subsequently transferred to a nitrocellulose membrane. Membranes were blocked with 5% TBST in skim milk for 1 h and incubated with anti-fibronectin antibody (#2413, Abcam) and/or anti-SA3N antibody (AF4709, R&D Systems, Minneapolis, MN, USA) at, respectively, a 1 : 1500 dilution and/or 1 : 700 dilution prepared in 5% TBST in skim milk and gently rocking overnight at 4 °C. Membranes were washed with TBST for 5 min, three times in total. After washes, membranes were incubated for 1 h with IRDye 800-conjugated anti-mouse and/or IRDye 700-conjugated anti-goat secondary immunoglobulin G antibody (both from Rockland Inc., Gilbertsville, PA, USA) at 1 : 5000 and/or 1 : 2000 dilutions and washed again with TBS for 5 min, three times in total. Subsequently, membranes were visualized with the Odyssey Infrared Imaging System (LI-COR Biotechnology, Lincoln, UK). Bands were detected and quantification of fibronectin densitometry was normalized to GAPDH when required.

### Statistical analyses

For the fibroblast detachment *in vitro* assays, one-way ANOVA with Dunnett's multiple comparison test was used for statistical analysis. For all other experiments, unpaired Student's *t*-test was used for statistical analyses. All statistical tests were performed using GraphPad Prism version 5.04 (GraphPad Software, San Diego, CA, USA). In all cases, *P*-values <0.05 were considered significant.

## Abbreviations

GzmB: granzyme B
ECM: extracellular matrix
SA3N: serpina3n
MMP: matrix metalloproteinase
AAA: abdominal aortic aneurysm
ACT: antichymotrypsin
PC: panniculus carnosus
*α*-SMA: alpha-smooth muscle actin
GAPDH: glyceraldehyde 3-phosphate dehydrogenase
